# The Selective Centrifugation Ensures a Better In Vitro Isolation of ASCs and Restores a Soft Tissue Regeneration In Vivo

**DOI:** 10.3390/ijms18051038

**Published:** 2017-05-12

**Authors:** Francesco De Francesco, Antonio Guastafierro, Gianfranco Nicoletti, Sergio Razzano, Michele Riccio, Giuseppe A. Ferraro

**Affiliations:** 1 Multidisciplinary Department of Medical-Surgical and Dental Specialties, Second University of Naples, Naples 80128, Italy; antonio.guastafierro.15@gmail.com (A.G.); giovannif.nicoletti@unina2.it (G.F.N.); razzanosergio@gmail.com (S.R.); giuseppe.ferraro@unina2.it (G.A.F.); 2 Department of Reconstructive Plastic Surgery-Hand Surgery, AOU “Ospedali Riuniti”, Ancona 60126, Italy; michelericcio.dr@gmail.com

**Keywords:** fat graft, adipose stem cells, regenerative surgery, breast reconstruction, pathological scar

## Abstract

Autologous fat grafting procedures in plastic surgery have been extensively used to reinforce soft tissue in congenital or acquired tissue impairments. With this background, the aim of this study is firstly to examine the impact of a selective centrifugation on existing adipose stem cells (ASCs) in terms of stemness profile maintenance and, secondly, to investigate the effect of restoring volume in reconstruction on patients affected by soft tissue damage. After centrifugation, the fat graft products were separated into two layers and subsequently examined in vitro for the expression of CD34, CD90, CD117, CD105, CD29, CD31, CD44, CD73, CD133, CD14 and CD45 markers by flow cytometry and gene expression analyses were performed for Sox2, WNT3A, END, CD44, FUT4, COLL1, CTNNB1, hbEGF, KRTLG, MMP2 and VIM genes. The results showed that in the middle-high density (MHD) layer there was a peak concentration of ASCs, compared to another layer obtained after centrifugation. Research carried out on patients under treatment for soft tissue regeneration using cells obtained from MHD layer selection will be fundamental in comparative analysis. These studies will lead to an adequate standardization of outcomes, provided that treatment is performed through cell selection. Therefore, a unique procedure in tissue reconstruction and regeneration through fat grafting is presented here.

## 1. Introduction

Fat grafting procedures are frequently used to restore volume and to regenerate tissues following the existence of stem cells in human adipose tissue, as revealed by Zuk et al. in 2002 [1]. Studies have shown that autologous fat grafting is effective in reconstructive and cosmetic treatments for patients affected by tissue volume loss due to aging, infection (facial lipoatrophy from HIV), trauma, congenital defects, in correcting breast defects following tumorectomy, and in post mastectomy reconstruction [2,3,4,5] or other soft tissue impairments generating asymmetry or deformity [6,7,8,9,10].

Adipose tissue contains adipocytes with a surrounding and supporting heterogeneous cell population known as stromal vascular fraction (SVF), which accommodates the adipose stem cells (ASCs) [11,12]. These cells possess self-renewal properties and may differentiate into multiple cell lineage properties [13]. Furthermore, ASCs are fundamental in regenerative treatments due to their abundance and low morbidity of harvesting qualities used specifically in soft tissue therapy to treat irradiated tissues, breast capsular contracture, damaged vocal cords, pathological scars, chronic wounds and perianal fistulas [14,15,16,17,18,19,20].

Autologous fat grafting, also known as lipofilling, is based on various techniques differing in harvesting centrifugation or infiltration methods. Fat grafting often provides favorable, albeit unpredictable long term outcomes. Some authors have attempted to minimize fat graft loss and to obtain a high rate of adipose stem cells that are easily administered into soft tissue, accessible for tissue regeneration. The purpose of this study is therefore to assess the stemness potential of cells within aspirated and centrifuged fat tissue, with particular attention paid to examining the molecular and cellular features of fat distributed cells after centrifugation [21]. Moreover, we showed the results regarding the clinical treatments of ASCs autografts using a selective centrifugation in a wide range of pathologies; this required volume restoration and soft tissue regeneration, which emphasizes the need to standardize procedures and technique conditions.

## 2. Results

### 2.1. In Vitro Experiments

**Figure 1:**
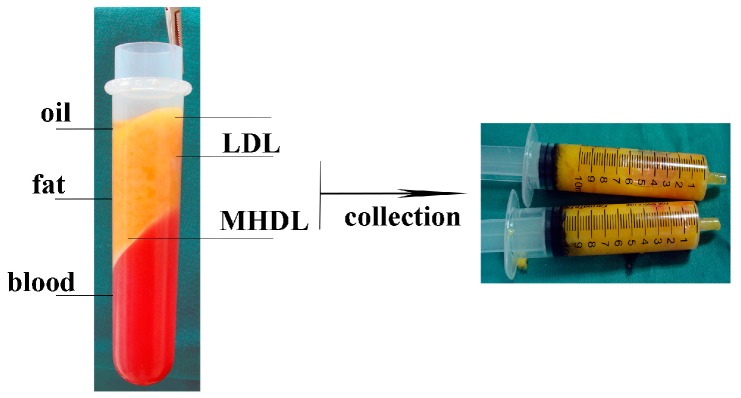
After centrifugation for 5 min at 1500 rpm, the fat sample is separated into three layers: an upper yellow layer of oil, a middle layer of adipose tissue, and a bottom layer of blood. The top and bottom layers are discarded. The middle layer is divided into two distinct layers: a low-density layer (LDL) and a middle-high-density layer (MHDL).

**Figure 2:**
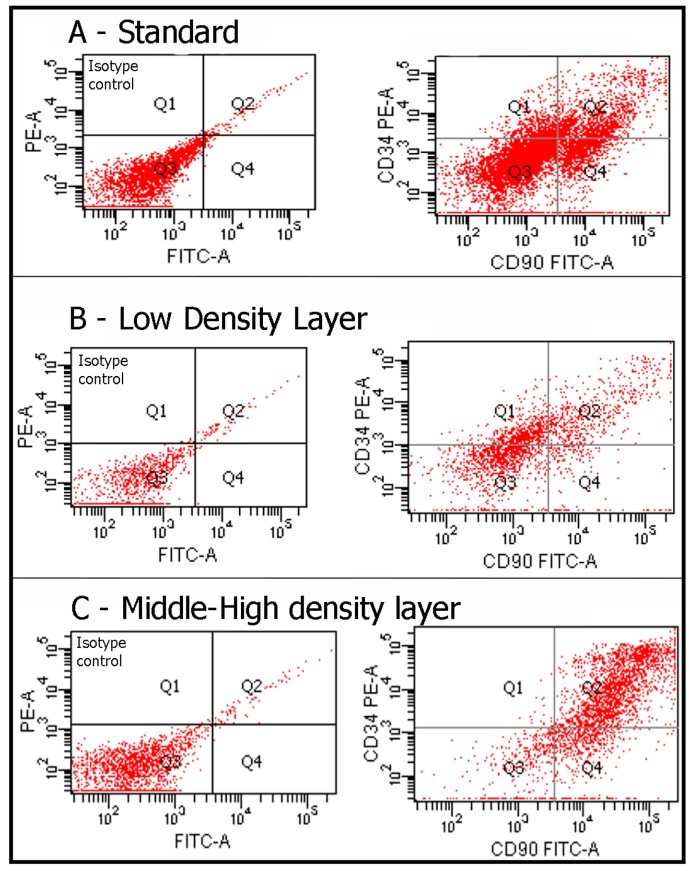
Flow cytometry analysis detected the presence of a cell population that we had previously identified [26,27,28,29]: the positive cells for these two markers were found in all layers. However, a greater number of positive CD34/CD90 cells was found in the MHDL compared to the LDL.

**Figure 3:**
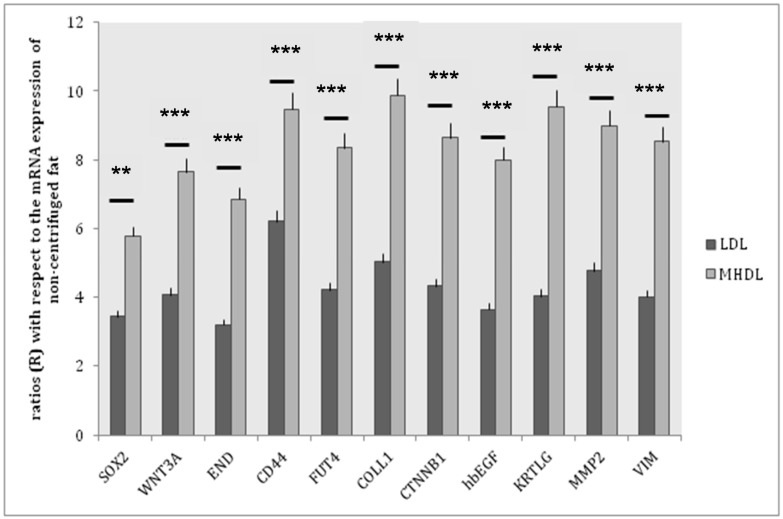
Gene expression of stemness markers in the two layers. Gene expression profile of Mesenchymal Stem Cell Specific Markers in LDL (white bars), MHDL (black bars) (*** *p* < 0.0005; ** *p* < 0.0025). The results are reported as ratios (R) with respect to the mRNA expression of non-centrifuged fat (not shown).

The fat of healthy patients was aspirated and centrifuged according to a technique, which separated the various components of the aspirated fat into three levels. The top level was the least dense and consisted mainly of oil. The central part was composed primarily of adipose tissue. The lower level was composed of blood, water, and other watery elements. The top and bottom layers were discarded. The middle layer (ML) was divided into two distinct part, as previously described by some authors [22,23]: a low density layer (LDL) and a middle-high density layer (MHDL) (Figure 1). We analyzed the phenotype of the cells in the samples by examining specific markers for mesenchymal stem cells. As we previously demonstrated in different papers, in accordance with the characterization criteria proposed by the International Society of Cellular Therapy (ISCT) and the International Fat Applied Technology Society (IFATS) [24,25], we detected stromal cells co-expressing CD117 and CD34 and mesenchymal stem cells, co-expressing CD29, CD34, CD44, CD90, CD73, CD105 and negative for CD31, CD14 and CD45 (data not shown, see references [26,27,28]) (Table 1 and Table 2). In particular, we focused our attention on a population of stem/progenitor cells expressing the following markers: CD34 and CD90. Such cell population has been previously and extensively studied in our earlier papers [27,29]: the positive cells for these two markers were found in all layers. However, in MHDL, cells showed a stronger positivity for CD34 and CD90 than those of cells in LDL (33% versus 12%) (Figure 2). In order to better characterize the stemness profile of cells within fat, the mRNA from each layer was extracted and analyzed using real-time PCR. The genes analyzed are described in Table 3. Stemness gene expression was detectable in the two layers. Non-centrifuged fat was used as the control. A significantly higher mRNA expression for all the markers related to stemness was detected in the MHDL compared to LDL confirming the distribution of stem cells in the fat subsequent to centrifugation. In particular, END, FUT4, COLL I, CTNNB1, KRTLG and VIM genes expression of cells derived from MHDL exhibited a two fold increase compared to those of cells derived from LDL (Figure 3).

### 2.2. In Vivo Experiment

#### 2.2.1. Breast Fat Grafting

**Figure 4:**
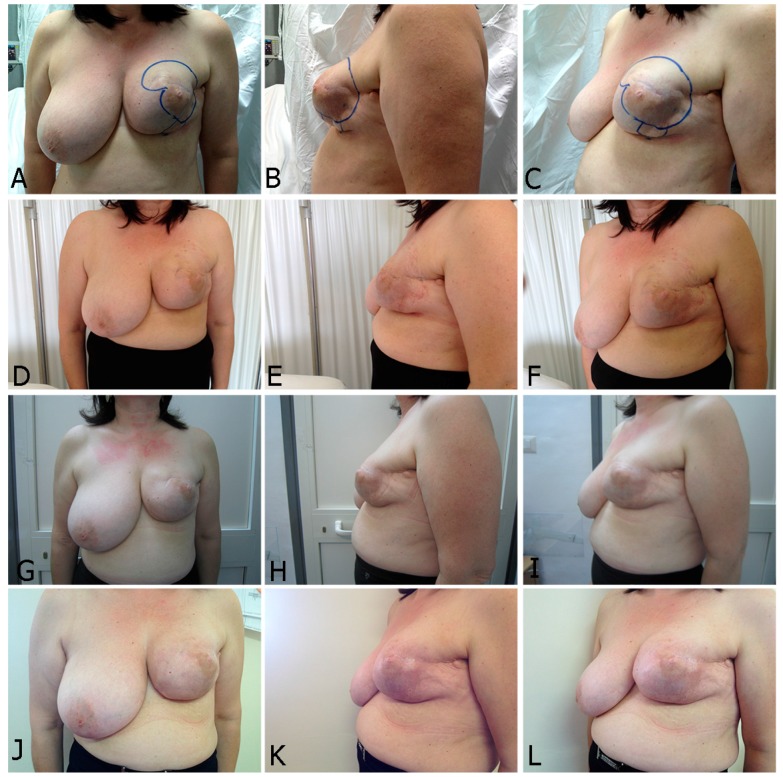
One case following stem cell therapy after a severe irradiation performed after expander insertion with dramatic capsular contracture with a high risk of exposure laterally (**A**–**C**). Two session of fat grafting performed (first: **D**–**F** and second: **G**–**I**). Stiffness and scarring were improved enormously (**J**–**L**). This follow-up is one year after the last treatment.

**Figure 5:**
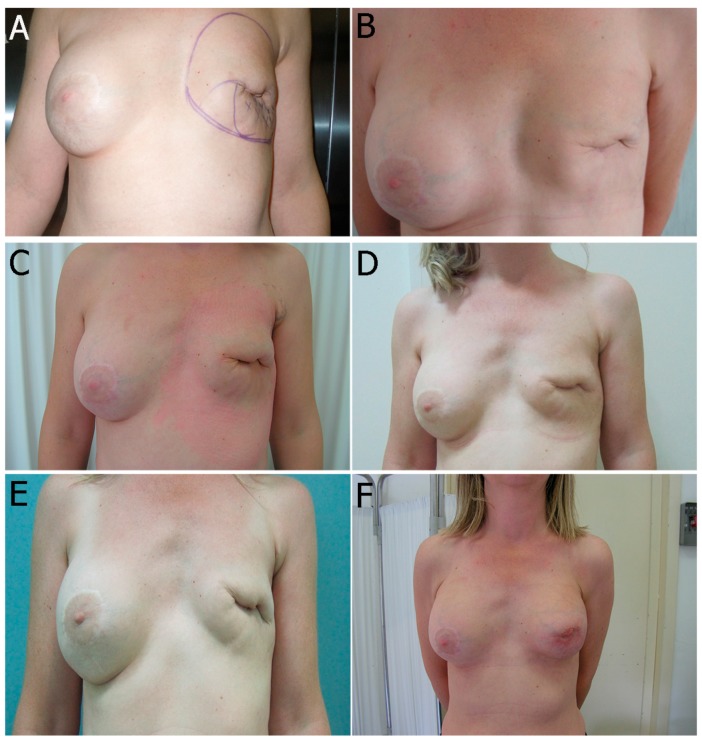
One case following stem cell therapy after a severe quadrantectomy irradiation and dramatic capsular contracture with prosthesis removal (**A**). Three sessions of fat grafting performed after prosthesis removal (**B**–**D**). After the last treatment, we inserted a new prosthesis and we performed the last fat-grafting treatment for aesthetic improvement (**E**,**F**). Stiffness and scarring were improved enormously. This follow-up is at two years.

A total of 25 female patients were operated on consecutively. The age of the patients ranged from 41 to 67 years (mean = 52.8 years). The mean number of sessions performed on each reconstructed breast was 3.4 ± 1.9 (range = 1–6 sessions) with an average volume of 245.5 ± 18 cc of autologous fat injected in each session (range = 200–300 cc). The average interval between two sessions was 3.9 ± 1.3 months (range = 2–6 months). The clinical outcome following fat grafting in mammary reconstruction included both volume increase and enhancement of regenerative potential. In all cases, the skin of the reconstructed or irradiated breast was supple, and the depression expansible, ideally with no evidence of post-radiation lesions such as telangiectasia or severe post-irradiation fibrosis. In addition, settlement of superficial irregularities and major pliability were observed. The aforementioned clinical benefits allow the rehabilitation of skin dystrophy, and are the results of regeneration on injection of ASCs layer (Table 4). Overcorrection was not performed in order to avoid presentation of irregularities of surface and contour, therefore minimizing risks of cystosteatonecrosis. After a follow-up period, the quadrantectomy/lumpectomy, the nipple-sparing mastectomy, the modified radical mastectomy, and post-irradiated cases presented no short-term (such as hematoma, infection, cellulitis) or long-term complications (Figure 4 and Figure 5).

**Figure 6:**
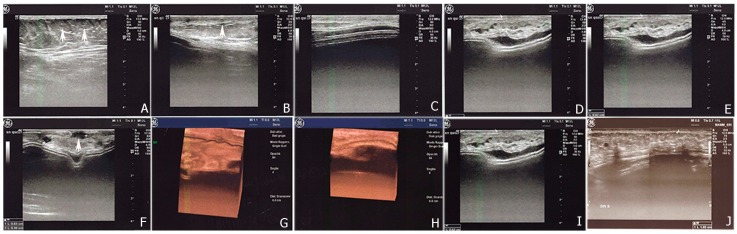
Ultrasonography figures showed a good visualization of the implanted adipose parenchyma showing internal echotexture inhomogeneous (**A**–**C**) and with the presence of small areolas hypo-anechoic with diameter max up to about 0.6 cm (**F**). 3D-4D scans show no structural alterations parenchyma (**G**,**H**). The prosthesis is discreetly located with irregular profiles and wavy characters without contracture, with the exception of some small areas characterized by thickening and splitting of the margin (**D**–**F**). The thickness of adipose tissue implanted is about 0.52 cm after lipofilling I and about 1.0 cm after the end of treatment (**I**,**J**).

**Figure 7:**
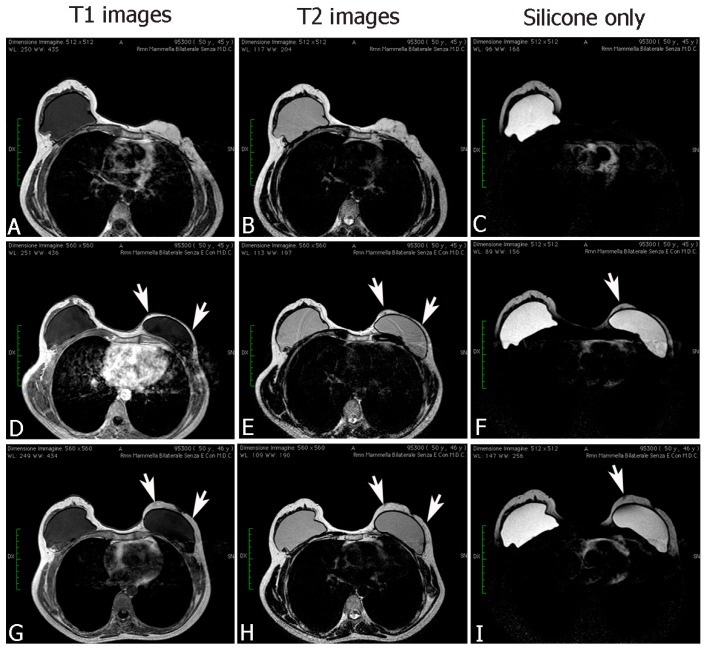
MRI figures, after fat transplantation to the breast, showed changes in volume, in thickness, and in fat distribution in the spaces (**G**–**I**) with respect to a previous treatment (**D**–**F**), and with respect to the pre-operative treatment (**A**–**C**). The fat grafting site was visible on MRI scans, with an identical signal of adjacent fat. The injected fat appearance is that of normal breast fat: hyperintense on T1- and T2-weighted sequences. On T1-weighted fat suppression images, the injected fat showed a hypointense normal fat signal. No signs of necrosis were presented on MRI.

Postoperative 2D/3D sonography (Figure 6) and MRI (Figure 7) identified changes in volume, in thickness, and in fat distribution in the spaces after autologous fat transplantation to the breast. Calcifying liponecrosis and oil cysts are conclusively diagnosed by ultrasonography and do not need further assessment. Microcalcifications indicating malignancy were not identified. MRI scans revealed the fat grafting section and corresponding signal of adjoining fat. The injected fat appearance as normal fatty breast tissue with hyperintense patches on the sequences in T1- and T2-weighted. The injected fat revealed a hypointense typical fat signal on the T1-weighted suppression sequences. No signs of necrosis were presented on MRI.

#### 2.2.2. Pathological Scar Fat Grafting

**Figure 8:**
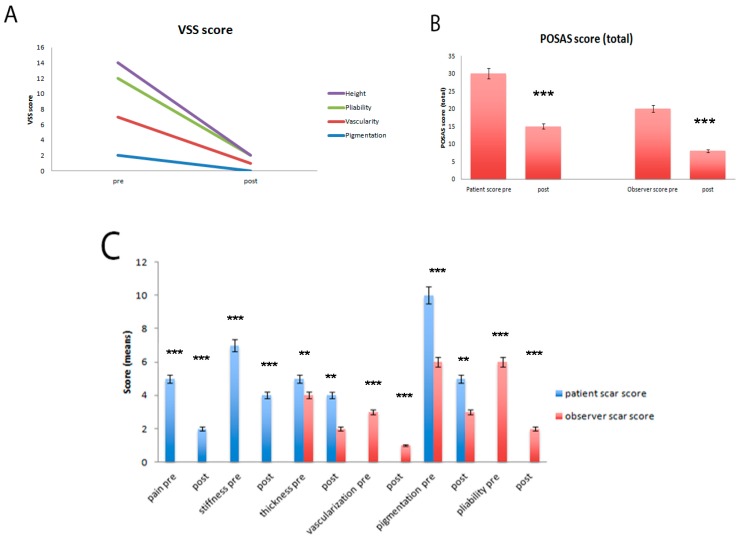
(**A**) Results of the patient and observer scar assessment scores at the final follow-up. The patient’s aesthetic and subjective complaints were significantly decreased for pain, pigmentation and stiffness (*p* = 0.0005). The observer’s scar scores were improved for all categories (*p* = 0.0003); (**B**) Total POSAS scores at the final follow-up. These improvements led to a significantly enhanced overall POSAS score in both the patient and observer *** *p* < 0.0005); (**C**) Total Vancouver scores at the final follow-up. These improvements led to a significantly enhanced overall VSS score in both the patient and observer (*** *p* < 0.0005; ** *p* < 0.0025).

**Figure 9:**
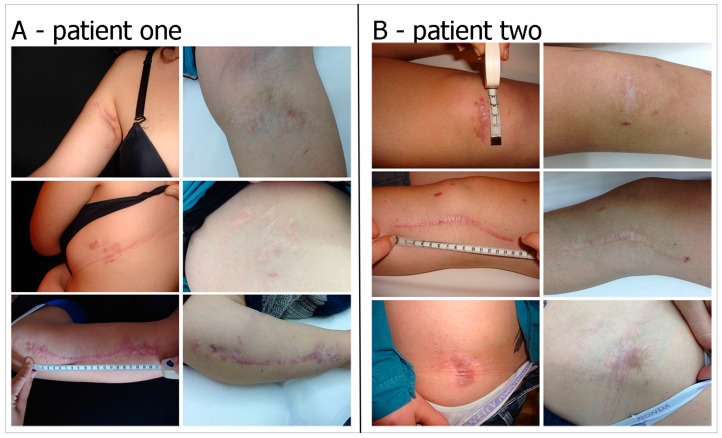
Two cases following stem cell therapy after posttraumatic scars. (**A**) Preoperative Posttraumatic scar on the left and postoperatively after one year of follow-up; (**B**) Preoperative Posttraumatic scar on the left and postoperatively after two years of follow-up.

**Figure 10:**
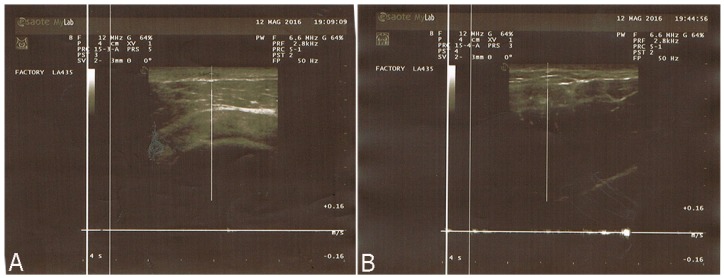
(**A**) Pre- and (**B**) postoperative laser Doppler spectroscopy measurements showed a reduction of microcirculation at the end of follow-up with a stabilization of scar remodeling.

A total of 15 patients were operated on consecutively. The age of the patients ranged from 42 to 67 years (mean = 54.8 years). All patients had previously undergone various unsuccessful treatments. The mean number of sessions performed on each pathological scar was 1.4 ± 0.9 (range = 1–3 sessions) with an average volume of 24.5 ± 10.8 cc of autologous fat injected in each session (range = 9–41 cc). The average interval between two sessions was 3.9 ± 1.3 months (range = 2–6 months). In the pathological scar group, the clinical outcome included, most importantly, a significant improvement in skin scar colour, pliability and texture. We observed, in all cases, a general enhancement in the quality of the scars, leading to improvements in the scar assessment scores assigned by both the observers and patients (Figure 8A). We observed, in particular, a relevant decrease in pain, stiffness and irregularity on termination of follow-up, in addition to a significant change in color, pigmentation and pliability (Figure 9A,B). These improvements led to a significantly enhanced overall POSAS and Vancouver score in all treated patients (Figure 8B,C). With regard to the microcirculation (Figure 10), we observed a reduction at the end of follow-up with a stabilization of scar remodeling. No cases of overcorrection, no cases of wound infections, skin necrosis, or loss of sensitivity were observed.

## 3. Discussion

Autologous fat grafting has several beneficial characteristics, such as lack of immunogenicity, simple surgical procedure, low cost, and easy accessibility, and has therefore become a popular technique for numerous reconstructive procedures [3,4,7,8,10] and aesthetic surgical treatments [6,30]. The grafting is composed of adipocytes and stromal vascular fraction cells, which specifically include ASCs. In particular, some studies regarding ASCs have recently gained significant attention due to their increased angiogenic capacity [27], differentiation property [29], and above all, their ability to adhere on biological substrates applicable in various clinical fields [28,31].

The aim of this study is to assess the different densities of ASCs within the injectable layers following centrifugation. Previous works have shown that the lower layer of the injectable fat yields the greatest density of viable cells [22,23]. Moreover, these results are correlated with an increased expression of mesenchymal stem cells markers such as LIF, SOX2, WNT3A, CTNNB1, FUT4, EGF, HGF, and VIM but also a high expression of endothelial markers and angiogenetic factors (such as VEGF), indicating significant vasculogenic properties of the cells in this layer [22]. In this study, we demonstrated that MHDL contained a higher percentage of cells expressing CD34 and CD90 than those of LDL. In previous studies, we have described that this cell subset showed a stemness profile in terms of proliferation, differentiation and higher telomerase activity. Here, we analyzed a series of genes involved in stemness such as SOX2, WNT3A, END, CTNNB1, VIM, FUT4, CD44, MMP2, KRTLG, CTNNB1 and COLL1 that were overexpressed in cells derived from MHDL, confirming the cytometric analyses. Therefore, the first result derived from experiments in vitro is that in MHDL is a concentrated population of mesenchymal stem cells. On the basis of these findings, we performed a clinical study employing the fat grafting obtained from the MHDL. In the mastectomy cases, the role of the fat grafting is used to treat the irradiated tissues to improve pliability, to release the mastectomy scar, to soften the scar and to increase the thickness of the subcutaneous tissue, restoring a tissue layer during an implant breast reconstruction between implant, muscles and skin. Our cases show the benefits of MHD layer fat grafting to the breast, such as its effectiveness in reverting radiation sequelae yielding aesthetic scars without donor site deformity, easily and at a low cost. To the best of our knowledge, there are few publications of a prospective and controlled study investigating the use of lipofilling breast volumetry in the field of breast reconstruction. Careful analysis of the literature highlights the presence of several approaches to fat graft differing in harvesting, centrifugation or infiltration methods [32,33,34,35,36]. In particular, some authors strongly support the potential of stem cells for the purpose of increasing fat graft survival, indicating the enrichment of lipofilling with stem cells as the best alternative to major tissue augmentation [37,38]. In contrast, other authors [39,40] support the role of improvement of recipient site as the endpoint of successful fat grafting. For this reason, Khouri defines this research as the “philosopher’s stone of fat grafting” [41], shifting the attention away from the preparation technique and towards the amount and vascularity of the receiving site. Oxygen delivery is crucial to achieving a good result, and this is possible by setting up an in situ biological scaffold that favors the survival of large volumes of autologous fat graft. Both ways of thinking are focused on one goal: the volumetric yield. However, our purpose is to show the regenerative potential of fat grafting, which is most likely due to the ability of cells to secrete various growth factors that improve survival and increase vascularization and consequently oxygenation. In this sense, our “multiple” sessions are aimed to regenerate tissues induced by ASCs with or without volumetric gain. In this context, Derby and colleagues [42] have demonstrated that ASCs increase collagen synthesis and vascularization through the production of growth factors after implantation, in vivo, improving dermal and skin tropism. This is based on our finding that aspirated fat tissue contains ASCs and these cells play a role in healing tissue damage. Moreover, ASCs are shown to survive in a low oxygen environment and to secrete angiogenic cytokines such as VEGF [27,43]. This suggests that the presence of ASCs improves fat graft viability and implantation and improves healed tissue, such as breast reconstruction sequelae and pathological scar, by stimulation of fibroblast proliferation, by secretion of type I collagen, and by increasing vessel density.

In these scenarios, this technique has also shown its clinical effectiveness in the treatment of atrophic retraent scars. In this clinical series, patients reported an important articular functional recovery in terms of range of motion, and a significant improvement of the skin, resulting in better pliability and less tension. Research has identified that cell inflammation is characteristic of fibrotic disease and may stimulate fibroblast development and expansion via cytokine promulgation [44]. ASCs are known to exert an anti-inflammatory action that regulates immune and inflammatory responses, providing therapeutic potential in various diseases. Clinically, the use of the MHD layer has proven its effectiveness in terms of improved texture, softness, thickness, color, and elasticity of the treated skin as well as a reduction of the scar retraction.

The methods and techniques used for grafting autologous adipose tissue remain varied with no evidence in literature of a unique procedure to process harvested fat. The fat grafting technique has improved over the years and has demonstrated that centrifugation at 1300 rpm for 5 min preserves the cell integrity and the characteristics of stemness, yielding minimal resorption rates [21]. Moreover, a further improvement of our technique regards the stemness concentration of ASCs in different layers of centrifuged fat [22]. The goal of our postharvested fat processing is to eliminate contaminants, including cellular debris, free oil, and other nonviable components of lipoaspirate such as hematogenous cells, as well as to maximize the number of adipose-derived mesenchymal stem cells in the grafted material towards greater graft viability. Moreover, ASCs in the MHD layer plays a fundamental role in regeneration and remodeling of damaged tissues, and it is to be considered an efficient instrument in tissue generation and regeneration. Our in vitro and in vivo results suggest that the replanting of the MHD layer obtained from lipoaspirate samples after centrifugation is sufficient to provide a significant number of ASCs and to preserve the adipocyte integrity, showing that both approaches are effective. The availability of adequate equipment to exploit the centrifugation technique may be a time-saving strategy in surgery.

## 4. Materials and Methods

### 4.1. In Vitro Experiments

#### Adipose Tissue Extraction and Digestion

Liposuction was performed in the Plastic and Reconstructive Surgery Clinic and adipose tissue was collected. The aspirate from the liposuction was set in NaCl 0.9%, and washed twice with phosphate-buffered saline (PBS: NaCl 137 mmol/L, KCl 2.7 mmol/L, Na_2_HPO_4_ 10 mmol/L, KH_2_PO_4_ 1.8 mmol/L), and centrifuged at 1300 rpm (250× *g*) for 5 min.

After centrifugation, specimens were divided into three portions, namely, oil (top), adipose (middle) and fluid (bottom). The adipose portion was divided equally into two layers: the lower two-thirds and the upper third (Figure 1). The two layers were placed, separately, in a solution of digestion containing collagenase type 1 (3 mg/mL), dispase (4 mg/mL) and supplemented penicillin (100 U/mL), streptomycin (100 g/mL), and clarythromicin (500 g/mL) in PBS at 37 °C, then agitated for 60 min. 70 μm filters (Becton & Dickinson, Sunnyvale, CA, USA) were used to filter the digestion solution.

### 4.2. Flow Cytometry

After digestion, at least 500,000 fresh cells were incubated with primary antibody for 30 min at 4 °C, washed twice in PBS and incubated with a secondary antibody. Antibodies used in this study were as follows: anti-CD117 PE (c-kit) (Miltenyi-Biotech, Calderara di Reno, Bologna, Italy—catalog number 130-098-212), anti-CD34 FITC and PE (Miltenyi-Biotech, Calderara di Reno, Bologna, Italy—catalog number 130-098-142 and 130-098-140), anti-CD90 FITC (BD Pharmigen, Buccinasco, Milan, Italy—catalog number 555595), anti-CD105 FITC (Santa Cruz, CA, USA—catalog number sc-18838), anti-CD29 Cy (Miltenyi-Biotech, Calderara di Reno, Bologna, Italy—catalog number 130-101-281), anti-CD31 FITC (Miltenyi-Biotech, Calderara di Reno, Bologna, Italy—catalog number 130-098-171), anti-CD133 PE (Miltenyi-Biotech, Calderara di Reno, Bologna, Italy—catalog number 130-098-826), anti-CD73 PE (BD Pharmigen, Buccinasco, Milan, Italy—catalog number 550257), anti-CD44 FITC (Miltenyi-Biotech, Calderara di Reno, Bologna, Italy—catalog number 130-098-210), anti-CD45 Cy and PE (BD Pharmigen, Buccinasco, Milan, Italy—catalog number 560974 and 555483), and anti-CD14 PE (Miltenyi-Biotech, Calderara di Reno, Bologna, Italy—catalog number 130-098-067). A FACS Vantage (Becton Dickinson) was used. CellQuest software was used for analyses.

### 4.3. Real-Time PCR

After digestion, the RNeasy Lipid Tissue kit (Qiagen, Milan, Italy) was used to obtain total RNA, as well as DNase digestion via RNase-Free DNase Set (Qiagen). Two different layers were obtained from the centrifuged lipoaspirate of the adipose tissue and from the lipoaspirate specimens that were not centrifuged. A RT2 First Strand kit (Qiagen) was utilized to reverse transcribe 800 ng of RNA. PCR in real time was carried out conforming to the instruction manual for the RT2 Profiler Human Mesenchymal Stem Cells PCR array (SABiosciences, Milan, Italy), and with SYBR Green ROX FAST Master Mix (Qiagen). A Rotor Gene Q 100 (Qiagen) was used to perform thermal cycling and to detect fluorescence. The data were examined with Excel-based PCR Array Data Analysis Templates (SABiosciences) and the results were recorded in ratios in relation to the mRNA expression of uncentrifuged fat.

### 4.4. In Vivo Experiments

#### Patients

Between March 2009 and March 2013, injections of autologous fat were administered to 40 patients. The inclusion criteria for the procedure were disease-free patients with close clinical follow-up, a minimum of eight months after completion of radiotherapy and the patient’s refusal to undergo autologous tissue reconstruction. Based on the inclusion criteria, a total of 25 patients were enrolled. In seven cases, lipoinjection was performed following quadrantectomy/lumpectomy, and radiation therapy. The remaining 18 patients underwent mastectomies as follows: ten patients received modified radical mastectomy with subsequent radiation treatment in two cases. Eight patients underwent nipple-sparing mastectomy with radiation treatment in one case. Of the 25 patients who underwent autologous fat grafting to improve the cosmetic result of reconstructive breast surgery, 10 patients had undergone parietal radiotherapy prior to breast reconstruction with implant. In these cases, fat injections were administered multidirectionally, from superficial to deep layers, spreading the fat throughout the irradiated area. In the modified radical mastectomy case, fat was transplanted in two different overlay planes in multiple tunnels. Following injection sessions, the final prosthesis was implanted under the pectoralis major muscle.

In 15 cases, the technique was used for treatment of post-traumatic scars. The injection phase was completed with small fat parcels implanted in multiple tunnels allowing full access of the fat graft to the surrounding blood supply.

We recorded the procedure date, the amount of injected fat, follow-up duration, and events of any complications that occurred. The pre-operative and post-operative images were analyzed by two masked surgeons who rated them according to the following rating scale: good/very good, moderate, or inadequate.

### 4.5. Surgical Technique

The patients were informed of standard preoperative instructions, including the use of antibiotics and recommendations against the use of aspirin and derivatives at least one week before surgery. For all the patients, the same technique of autologous fat grafting was performed. Tumescent injection was used (we used 1:200,000 epinephrine injected through a 22-gauge spinal needle before aspiration). The fat donor sites were the hips, the abdomen, the trochanteric area, and the inner sides of the thigh and knees. The fat was collected using a two-holed blunt harvesting cannula (3-mm inner diameter) attached to a 10-mL Luer-Lok syringe (Becton Dickinson, Franklin Lakes, NJ, USA), progressively applying a gentle negative pressure and pulling gradually back on the plunger. The capped 10-mL Luer-Lok syringes were placed in a centrifuge and spun at 1300 rpm (250× *g*) for 5 min. Following the centrifugation procedure, three layers were obtained from the specimens: an oily yellow top layer acquired from the destroyed fatty tissue fragments, a central layer of adipose tissue graft, and a bottom layer composed of blood. After discarding the oil (superiorly) and the blood (inferiorly), we collected the middle layer and in particular, due to the short oil derived residual properties, we selected the lower two-thirds of purified lipoaspirate deposited in the lower part of the syringe near the needle after the removal of red cells.

The selected lipoaspirate was transferred through a Luer-Lok connector to 3-mL syringes and injected with a 17-G one-holed blunt cannula in a multilayer, multidirectional manner in the entire regenerative area.

Follow-up examinations and imaging techniques were used for evaluation of fat graft viability of twenty-five patients undergoing autologous fat grating to improve cosmetic outcomes of reconstructive breast surgery; patients were subjected to preoperative and postoperative ultrasound in 2D/3D and magnetic resonance imaging of the breast to establish volume, thickness and fat distribution in the spaces.

For 15 patients who underwent autologous fat grafting to improve the cosmetic result of pathological scars, the pre- and postoperative examination included use of the POSAS (Patient and Observer Scar Assessment Scale), Vancouver Scale and photo documentation, as well as the measurement of microcirculation using the Doppler ultrasound.

### 4.6. Statistical Analysis

All data were statistically analyzed using One-way ANOVA test. The POSAS and Vancouver score results were compared using an unpaired *t*-test. The threshold for statistical significance was established at *p*-values < 0.05. Repeatability was considered as a standard deviation to calculate differences between measurements using SPSS 16.0 software (SPSS Inc., Chicago, IL, USA) for testing analysis.

### 4.7. Ethics Statement

The study respects all ethical requirements in its objectives and methodologies. We strictly comply with widely recognized international codes of practice such as the Nuremberg code, the Helsinki agreement, the conventions of the Council of Europe on human rights and biomedicine, with particularly attention to EU legislation: 2001/83/EC, 86/609/EEC and FP7 Decision nr 1982/2006EC. Human biological samples are necessary because we need to test human cells, which have unique biological characteristics, distinct from those of animals. The overall intention in the project is to reduce the number of animal experiments. Only adult patients who are able to give consent will be included. All the patients that are the subjects of our study donated their consensus to scientific treatment and publication of their clinic situation and images. We have obtained written informed consent from all patients. This study was approved by our Internal Ethical Committee (RegStem01–03/2009) without any registration in public registry because this study is not a clinical trial.

## 5. Conclusions

In conclusion, there are several ways to optimize a fat grafting procedure and our study has provided evidence that stem cells selected from the middle-high density layer of human adipose tissue, after centrifugation, constitute a large, ideal source of cells for tissue regeneration, and tissue-based clinical therapies. However, other techniques may be concurrently used, including PRP [45] or Rigenera protocol [46,47]. A better understanding of fat grafting highlights its regenerative potential in terms of applicability, cost and effectiveness, and its ability to reduce lengthy and expensive treatments.

## Figures and Tables

**Table 1:** Mean percentage of surface markers expression in both groups (MHDL (middle-high density layer) and LDL (low density layer)).

**MHDL Group**												
	CD14 (%)	CD29 (%)	CD31 (%)	CD34 (%)	CD44 (%)	CD45 (%)	CD73 (%)	CD90 (%)	CD105 (%)	CD117 (%)	CD133 (%)	CD34/CD90 (%)
*n*	40	40	40	40	40	40	40	40	40	40	40	40
Average	0%	42%	6%	33%	5%	0%	45%	62%	28%	10%	8%	85%
Minimum	0%	26%	4%	23%	2%	0%	37%	53%	13%	3%	4%	78%
Maximum	0%	48%	10%	38%	8%	0%	53%	76%	34%	16%	14%	90%
**LDL Group**												
*n*	40	40	40	40	40	40	40	40	40	40	40	40
Average	0%	15%	3%	12%	2%	0%	20%	31%	12%	4%	6%	63%
Minimum	0%	10%	1%	8%	1%	0%	10%	23%	7%	1%	2%	59%
Maximum	0%	25%	5%	20%	5%	0%	40%	44%	26%	8%	10%	65%

**Table 2:** Statistical analyses of markers distribution in MHDL and LDL groups.

Cluster of Differentiation	MHDL Group (%)	LDL Group (%)	*p*-Value
CD14	0	0	
CD29	42	15	*p* < 0.001
CD31	6	3	*p* = 0.005
CD34	33	12	*p* < 0.0005
CD44	5	2	*p* = 0.005
CD45	0	0	
CD73	45	20	*p* < 0.0005
CD90	62	31	*p* < 0.0005
CD105	28	12	*p* < 0.001
CD117	10	4	*p* < 0.001
CD133	8	6	*p* = 0.005
CD34+CD90+	85	63	*p* < 0.0005

**Table 3:** In order to characterize the stemness properties of cells within fat, the mRNA from each layer was extracted and analyzed by real-time PCR.

Gene	Function
*SOX2*	*SOX2* is a transcriptional activator that identity neural stem cells. In addition to regulating the progression of neurogenesis, this group of activators is also active in post-mitotic neurons.
*WNT3A*	WNTs are a family of lipid-modified secreted glycoproteins involved on stem cell niches.
*END*	(endoglin or CD 105): human endoglin is an RGD-containing transmembrane glycoprotein identified in vascular endothelial cells.
*CD44*	A ubiquitous multistructural and multifunctional cell surface adhesion molecule involved in cell-cell and cell-matrix interactions. CD44 also participates in the uptake and intracellular degradation of HA, it is present in mesenchymal stem cells.
*FUT4*	A myeloid α1,3-fucosyltransferase responsible for the fucosylation of adhesive interactions between selectins and their ligands, and it plays an essential role in hematopoietic cell lines.
*COLL1*	Collagen type I is present in the extracellular matrix and it is produced by mesenchymal stem cells.
*CTNNB1*	The *β-catenin* gene is involved in WNT signaling. The WNT factor family plays numerous roles in embryonic development and stem cell biology. WNT signaling is transduced by the FZD family of receptors.
*hbEGF*	Heparin-binding epidermal growth factor-like growth factor: a member of the EGF family of growth factors, which interact with the EGF receptor to exert mitogenic activity in various cell types. Recent studies indicate that HB-EGF contributes to neuronal survival and the proliferation of glial/stem cells.
*KRTLG*	Also know as Stem Cell Factor (SCF): a dimeric molecule that exerts its biological functions by binding to and activating the receptor tyrosine kinase c-Kit. Signaling from c-Kit is crucial for normal hematopoiesis, pigmentation, fertility, gut motility, and some aspects of the nervous system.
*MMP2*	Matrix metalloproteinases 2: a protein involved in the migration of hematopoietic stem cells through the blood and across the endothelial vasculature to different organs and to their bone marrow (BM) niches, or, in other words, during the homing process.
*VIM*	Vimentin: a type III intermediate filament protein that is expressed in mesenchymal cells.

**Table 4:** Clinical summary of treated breasts and clinical outcomes.

**Patients Characteristics**		
No. of cases	25	
Sex	25 Female, 0 Male	
Age (years)	52.8	
Surgical procedure *		
group A group B group C	7 cases 10 cases 8 cases	
Radiotherapy (RT)	10 cases	
Site of liposuctions		
Thights Thights and abdomen	10 cases 15 cases	
Number of section	3.4 ± 1.9	
Average volume	245.5 ± 18 cc	
Interval between sessions	3.9 ± 1.3 months	
	**Clinical Assessment before Treatment**	**Clinical Assessment Post Fat Grafting**
RT	Fibrosis, atrophy, retraction, ulcers, ulcers with implant exposure, telangectasia, itching	Remission of symptoms of RT therapy
No-RT **		
Volume (respect controlateral)		4
Contour		5
Breast implant (contracture, wrinkling, extrusion)		4
Pliability		5
Thickness		5
Overall result		23

* Group A: quadrantectomy/lumpectomy; Group B: radical mastectomy; Group C: nipple-sparing mastectomy. ** The evaluation method was based on six different items with different Likert subscales. The cumulative score (0–25) served to classify the overall results as follow: 0–12 as bad, 13–16 as poor, 17–20 as fair, 21–22 as good, 23–24 as very good and 25 as excellent.
